# A Sequalae of Lineage Divergence in *Staphylococcus aureus* from Community-Acquired Patterns in Youth to Hospital-Associated Profiles in Seniors Implied Age-Specific Host-Selection from a Common Ancestor

**DOI:** 10.3390/diagnostics13050819

**Published:** 2023-02-21

**Authors:** Kamaleldin B. Said, Naif Saad AlGhasab, Mohammed S. M. Alharbi, Ahmed Alsolami, Abdelhafiz I. Bashir, Mohd Saleem, Azharuddin Sajid Syed Khaja, Dakheel F. Aldakheel, Ehab Rakha, Jabar A. Alshamri, Awdah Al-hazimi, Adel J. Alrodhaiman, Taha E. Taha, Hamad H. Alanazi, Ha’il COM Research Unit Group

**Affiliations:** 1 Department of Pathology, College of Medicine, University of Ha’il, Ha’il 55476, Saudi Arabia; 2 Genomics, Bioinformatics and Systems Biology, Carleton University, 1125 Colonel-By Drive, Ottawa, ON K1S 5B6, Canada; 3 Department of Cardiology, College of Medicine, University of Ha’il, Ha’il 55476, Saudi Arabia; 4 Department of Internal Medicine, College of Medicine, University of Ha’il, Ha’il 55476, Saudi Arabia; 5 Department of Physiology, College of Medicine, University of Hail, Ha’il 55476, Saudi Arabia; 6 Medical Coordination Unit, Ha’il General Hospital, Ha’il 55428, Saudi Arabia; 7 Departments of Microbiology, King Khalid Hospital, Ha’il 55421, Saudi Arabia; 8 Clinical Pathology Department, Faculty of Medicine, Mansoura University, Mansoura 35516, Egypt; 9 Department of Training and Education, King Khalid Hospital, Ha’il 55421, Saudi Arabia; 10 Department of Epidemiology, John Hopkins Bloomberg School of Public Health, Baltimore, MD 21205, USA

**Keywords:** CA-MRSA, HA-MRSA, nosocomial *S. aureus*, *S. aureus* epidemiology

## Abstract

The rapidly changing epidemiology of *Staphylococcus aureus* and evolution of strains with enhanced virulence is a significant issue in global healthcare. Hospital-associated methicillin-resistant *S. aureus* (HA-MRSA) lineages are being completely replaced by community-associated *S. aureus* (CA-MRSA) in many regions. Surveillance programs tracing the reservoirs and sources of infections are needed. Using molecular diagnostics, antibiograms, and patient demographics, we have examined the distributions of *S. aureus* in Ha’il hospitals. Out of 274 *S. aureus* isolates recovered from clinical specimens, 181 (66%, *n* = 181) were MRSA, some with HA-MRSA patterns across 26 antimicrobials with almost full resistances to all beta-lactams, while the majority were highly susceptible to all non-beta-lactams, indicating the CA-MRSA type. The rest of isolates (34%, *n* = 93) were methicillin-susceptible, penicillin-resistant MSSA lineages (90%). The MRSA in men was over 56% among total MRSA (*n* = 181) isolates and 37% of overall isolates (*n* = 102 of 274) compared to MSSA in total isolates (17.5%, *n* = 48), respectively. However, these were 28.4% (*n* = 78) and 12.4% (*n* = 34) for MRSA and MSSA infections in women, respectively. MRSA rates per age groups of 0–20, 21–50, and >50 years of age were 15% (*n* = 42), 17% (*n* = 48), and 32% (*n* = 89), respectively. However, MSSA in the same age groups were 13% (*n* = 35), 9% (*n* = 25), and 8% (*n* = 22). Interestingly, MRSA increased proportional to age, while MSSA concomitantly decreased, implying dominance of the latter ancestors early in life and then gradual replacement by MRSA. The dominance and seriousness of MRSA despite enormous efforts in place is potentially for the increased use of beta-lactams known to enhance virulence. The Intriguing prevalence of the CA-MRSA patterns in young otherwise healthy individuals replaced by MRSA later in seniors and the dominance of penicillin-resistant MSSA phenotypes imply three types of host- and age-specific evolutionary lineages. Thus, the decreasing MSSA trend by age with concomitant increase and sub-clonal differentiation into HA-MRSA in seniors and CA-MRSA in young and otherwise healthy patients strongly support the notion of subclinal emergences from a resident penicillin-resistant MSSA ancestor. Future vertical studies should focus on the surveillance of invasive CA-MRSA rates and phenotypes.

## 1. Introduction

*Staphylococcus aureus* is one of the leading causes of skin and soft tissue infections that are either uncomplicated, severe, or invasive in nature [1,2,3]. It is also a leading bacterial agent in septic pneumonia and other respiratory tract, surgical site, prosthetic joint, and cardiovascular infections, all leading to severe nosocomial bacteremia [4]. As such, *S. aureus* is the most dominant Gram-positive species that is specifically adapted to humans as commensals that rapidly turns into deadly pathogens in various clinical diseases. It precisely inhabits the human skin and anterior nares such as the axillae and groin with around ~20% of nasal colonization in different populations [1]. *S. aureus* typically causes a wide range of skin infections, including impetigo, skin abscess, furuncles, wound infections, and septic shock syndrome [5]. This wide range of diseases is due to the large and variable arsenals of virulence factors such as surface proteins, degradative enzymes, cytotoxins, biofilms formations, and antibiotic resistance genes. This is in addition to an array of differential expression profiles of intrinsic chromosomal genes that are turned on by host microenvironmental conditions [6]. Thus, surveillance of infections becomes imperative for the distribution and frequency of *S. aureus* infections in hospitals.

One of the major reasons for the seriousness of *S. aureus* infections is the widely spread methicillin-resistant *S. aureus* (MRSA) lineages causing increased mortality, morbidity, and hospital stays, as compared to methicillin-sensitive *S. aureus* (MSSA) lineages [7]. Balkhy et al. (2016) [8] reported on the strategic planning of the Gulf Cooperation Council Center for Infection Control (GCC-IC) that has placed the emergence of antimicrobial resistance (AMR) on the top of its agenda since 2014. This was soon followed by the second round table discussion on the “One Health” concept from the Gulf Cooperation Council Countries (“Part Two: A Focus on Human Health”) [9]. Nevertheless, despite enormous efforts, and as is the case globally, calls for regional MRSA surveillance programs were made especially with the emergence of strains that require no underlying risk factors to cause illness, as well as the propagation of chimeric resistance elements in both HA-MRSA and CA-MRSA [10].^.^ This preceded several reports on the rise of *S. aureus* resistant strains in the region. Most of the MRSA isolates were predominantly from skin, soft tissue, wounds, and nasal swabs [6,11,12,13,14,15]. The antibiotics most commonly used for MRSA infections (skin and soft tissue infection) included fusidic acid, mupirocin, vancomycin, and clindamycin. Most resistances were found in the eastern region of Saudi Arabia compared to Riyadh and other cities [6,16,17], while resistance has increased by three times the usual in the UAE and Gulf countries, which suffered higher rates of resistance [17,18,19,20,21].

The origin of most deadly infections of *S. aureus* is a simple skin inhabitant variant of this species. The success of this highly contagious pathogen on skin and soft tissue infections depends on the elaborate adaptive mechanisms of virulence and immune evasion of host defenses. These include but are not limited to the local adaptive response to treatment and control options: for instance, production of specific toxins that destroy phagocytes such as leucocidins, which trigger phagocyte apoptosis, inhibition of complement factors, and inhibition of agglutination and the formation of thrombi. This way the resident skin *S. aureus* turns into a deadly pathogen in a four-stage mechanism bypassing initial immune response and neutrophils and surviving in blood, followed by abscess formation in few days, and finally launching a severe attack where variants mutate to persist [22,23]. The result of this is the emergence of highly successful strain variant from a skin reservoir of existing ones. For instance, MRSA genotypes with unique virulence, including a high prevalence of PVL and fusidic acid resistance in Kuwait hospitals was reported [24,25,26,27]. To avoid host immunity, *S. aureus* occupies intracellular shelter within host cells such as the phagocytes neutrophils and monocytes [28,29], as well as a series of non-phagocytic cells including epithelial and endothelial cells, keratinocytes, and osteoblasts [30]. Persistent in *S. aureus* in neutrophils is a virulence mechanism that serves as a reservoir leading to chronic as well as acute infections [31]. The rapid adaptational changes and strain differentiation into virulence come from genome plasticity, transfer of resistance, and an array of gene subsets in an accessory genome making this species one of the most contagious human-associated pathogens in history. This makes it imperative for multipoint local surveillance programs to establish rigid strategic control plans.

Methicillin-resistant *Staphylococcus aureus* is subdivided into lineages based on molecular typing (sequence type STs), genomic, and other data. There are a large number of new lineages that replaced old ones. Of particular concern is the progressive global replacement of hospital-associated MRSA (HA-MRSA) by community-associated MRSA (CA-MRSA) lineages. It has been widely known that MRSA was initially confined to hospitals and resistant to almost all types of beta-lactams; whereas, CA-MRSA is known to carry Panton–Valentine leucocidin and is susceptible to non-beta-lactams [32].The CA-MRSA lineages are well known for their rapid community transmission, aggressive skin and soft tissue infections, and severe community-acquired necrotizing pneumonia. These lineages were top listed as significant threats since the previous MRSA pandemic that caused mortality rates similar to that of AIDS, tuberculosis, and viral hepatitis combined [2,33,34,35,36]. At present, the evolution and emergence of invasive lineages of CA-MRSA, HA-MRSA, as well as MSSA causing bloodstream infections are being increasingly reported, leading to changes in global clonal profiles [7,37] with complete replacement of HA-MRSA by CA-MRSA occasionally in some cases. In China, since 2013, CA-MRSA strains have included ST59 largely replacing HA-MRSA ST239 [38]. This has also been the case in South Asia [39], in Africa [40], Australia, USA [41], India [42], and Canada [43].

The evolutionary lines of MRSA lineages have not been clear in the Middle Eastern countries. It has been shown that the European CC1-MRSA-IV appeared around 1995 and was widespread throughout Europe and into the Middle East [44]. Since then, the evolutionary lines of these lineages have not been clear and have been further complicated by the appearance of livestock lineages such as bovine mastitis lineage poultry lineage, and food-associated lineages in Arab countries [45]. The World Health Organization (WHO) labeled MRSA as one of the indicators for antimicrobial resistance in the Sustainable Development Goals connected to the health target 3.d [46]. Furthermore, the emerging MRSA lineages have become critically important in pediatrics targeting otherwise healthy young individuals. However, the rates, frequencies, and distribution of *S. aureus* lineages in hospitals are not adequately addressed owing to the paucity in high quality data on local strain profiles and infection patterns. Thus, this study aims to investigate the molecular profiles, antibiograms, and age- and gender-specific distributions of *S. aureus* lineages isolated from cutaneous infections and to establish a precise understanding of skin carriage as a source of transmission dynamics.

## 2. Materials and Methods

### 2.1. Microbiological Diagnosis, Antimicrobial Susceptibility Testing, and Patients’ Demographics

Microbiological Diagnosis, antimicrobial susceptibility, and patient demographic data were all obtained from laboratory records, hospital medical records, and other sources in the hospitals. All records of clinical specimens from different hospital departments were processed for selection of non-duplicate isolates of *Staphylococcus aureus* from hospitals in Ha’il from September to December 2021. These were then subjected to subsequent diagnostic characterizations into methicillin-resistant *S. aureus* (MRSA) and methicillin-susceptible *S. aureus* (MSSA) isolates. For this, standard microbiological analysis and antimicrobial sensitivity testing, followed by molecular profiling of lineages, were conducted as follows.

For non-automated routine protocols, specimens were aseptically and professionally collected in suitable transport media, swabs, and/or media, send to the lab, and processed immediately or cultured for primary identifications using standard conditions and media incubated at 37 °C incubations for at least 18 h. Stock cultures of bacterial isolates were immediately kept in broth media at −80 °C for future reference and vertical studies. For automated protocols, specimens or cultures were concomitantly prepared and used for identification by automated testing and ID susceptibility testing using automated systems. Most the this phase was performed on the BD Phoenix system (BD Biosciences, Franklin Lakes, NJ, USA) and MicroScan plus (Beckman Coulter, Brea, CA, USA). When required, sensitivities were confirmed by in vitro cultures in agar diffusions interpreted by zone interpretive standards for this region. The susceptibility testing and breakpoint interpretive standards were carried out in accordance with the recommendations of the Clinical and Laboratory Standard Institute (CLSI document M100S-26) [47].

### 2.2. Resistance Classifications of MRSA Lineages Based on Standard Definitions for Classification: As Multi-Drug Resistant Bacteria (MDR)

Staphylococcus acquired resistance classifications are based on standard definitions for classifications that considers MRSA isolates as multi drug-resistant (MDR) for their methicillin resistance and resistance to beta-lactams except for the community-acquired lineages (CA-MRSA), since they are susceptible to beta-lactams. These definitions are according to the recommendations of the CDC and European Centre for Disease Control. The following definitions are usually accepted as standard according to Magiorakos et al., [48]:

MDR = non-susceptibility to at least one agent in three or more antimicrobial categories;
XDR = non-susceptibility to at least one agent in all but two or fewer antimicrobial categories (i.e., bacterial isolates remain susceptible to only one or two categories);
PDR = non-susceptibility to all agents in all antimicrobial categories as reported.

We did not include known intrinsic resistances to particular drugs. Thus, MRSA criteria for defining *S. aureus* MDR classifications must include one or more of the following to apply: 1. hospital-acquired MRSA is always considered MDR by virtue of being an MRSA; 2. non-susceptible to ≥1 agent in >3 antimicrobial categories.

### 2.3. Molecular Detection and Characterization of S. aureus Lineages by Multi-Gene GeneXpert System

Simultaneous confirmation, identification, and molecular profiling of *S. aureus* directly from the specimen is carried out using the latest versions of the Cepheid GeneXpert^®^ Dx system with specific all-in-one cartilages of the SA Complete and MRSA assay kits, following manufacturers’ recommendations and names and codes included in each kit. The system consists of an instrument, personal computer, and preloaded software for running tests and viewing the results. Depending on the kit used, assay definition files are imported into the software, such as the Blood Culture assay definition file. To start, when the computer is turned on the GeneXpert lunches automatically or is clicked to start, then log-on, create test, or orders. In the following steps sample patient IDs are scanned, and kit barcodes will re-populate boxes with Assay, Reagent Lot ID, Cartridge SN, and Expiration Date. Although some specimens like sputum require a few simple steps such as homogenizations and mixing before loading into cartridges, the general protocol for all kits is concise, automated, user-friendly, and highly robust. For example, Xpert MRSA/SA SSTI (skin and soft-tissue infections, as well as wounds, surgical infections) swabs from deep tissue, surgical site and wound infections were inserted into the sample reagent vial (break-in). Then, vortex well, particularly for samples with mucus contents or debris, dispense the sample into Port-S, insert the cartridge, and start the test. Multi-gene primers, probes, and reagents in kits allow for robust automated direct confirmation of *S. aureus* at species level with subsequent differentiation into *S. aureus* lineages directly from specimens. This is accomplished by the built-in primers for nuc spa, mecA and the mec (SCCmec) gene direct detections from specimens utilizing automated real-time polymerase chain reaction (PCR) in a single-use, disposable, self-contained cartridge with PCR reagents inserted and inoculated directly with swabs/samples. This method allows for minimizing laboratory media influence on *S. aureus* that is known to trigger sensing genes, leading to adaptive genome expressions and the emergence of different types. In addition, it reduces cross-contamination between specimens as well as cross-sequence contaminations in molecular tests. These are all remote since the cartridge is a disposable, closed, and self-contained kit. Furthermore, a sample processing control (SPC) and a probe check control (PCC) are also included. The SPC is present to control for adequate processing of the target bacteria and to monitor the presence of inhibitor(s) in the PCR reaction. The PCC verifies reagent rehydration, PCR tube filling in the cartridge, probe integrity, and dye stability.

### 2.4. Statistical Analysis

Collected data were analyzed using Statistical Package for Social Sciences software (IBM SPSS; Version 24 SPSS version 23.0 for Windows (SPSS, Inc., Chicago, IL, USA). Descriptive and stratified analysis were conducted; we present absolute numbers, proportions, and graphical distributions. We conducted exact statistical tests for proportions and show *p*-values (based on Chi square test values) where appropriate (a *p*-value < 0.05 was considered statistically significant).

## 3. Results

**Figure 1:**
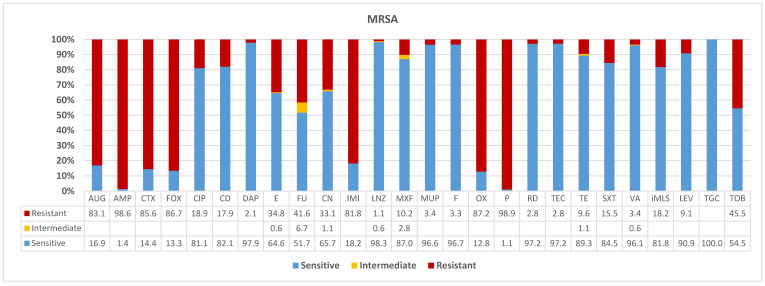
**Antibiogram patterns of methicillin-resistant *Staphylococcus aureus* isolates recovered from clinical specimens in Ha’il region, Saudi Arabia.** AUG—amoxicillin */clavulanic acid (2/1); AMP—ampicillin, CTX—cefotaxime; FOX—cefoxitin, CIP—ciprofloxacin; CD—clindamycin; DAP—daptomycin, E—erythromycin; FU—fusidic acid: CN—gentamicin; IMI—imipenem, LNZ—linezolid; MXF—moxifloxacin; MUP—mupirocin; F—Nitrofuran; OX—oxacillin; P—penicillin; RD—rifampicin; TEC—teicoplanin; TE—tetracycline; SXT—trimethoprim */sulfamethoxazole; VA—vancomycin; IMLS—inducible macrolide, lincosamide, and streptogramin; LEV—levofloxacin; TGC—tigecycline; TOB—tobramycin.

In this study, 276 *S. aureus* isolates were recovered from positive specimens in skin related infections. All isolates were identified and characterized as methicillin-sensitive *S. aureus* and methicillin-resistant *S. aureus* lineages by molecular markers and antimicrobial resistance determinations. Here, we show that lineages were uniquely associated to different age- and gender-specific groups of patients in the hospital. Of the isolates analyzed, 183 were MRSA showing different resistance patterns across 26 antimicrobials. For instance, as shown in Figure 1 and Table 1, for seven antimicrobials, MRSA isolates showed very high resistances including, IMI (82%), Aug (83%), CTX (85.6%), FOX (86.7%), OX (87%), and almost full resistances to P (99%) and AMP (98.6%). However, for several antimicrobial classes high susceptibility was seen often reaching to full susceptibility. These were: TGC (100%), RD (97.2%), TEC (97.2%), VA (96%), LEV (91%), TE (89.3%), SXT (84.5%), iMLS (81%).

**Figure 2:**
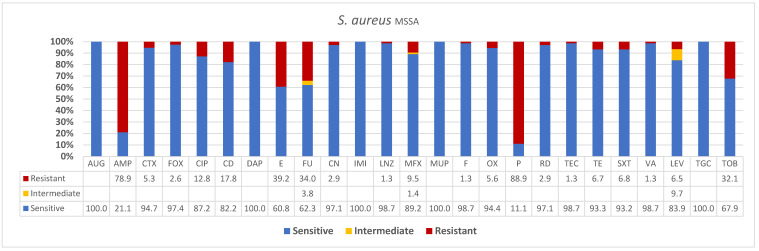
**Antibiogram patterns of methicillin-susceptible *Staphylococcus aureus* isolates recovered from clinical specimens in Ha’il region, Saudi Arabia** AUG—amoxicillin */clavulanic acid (2/1), AMP—ampicillin, CTX—cefotaxime, FOX—cefoxitin, CIP—ciprofloxacin, CD—clindamycin, DAP—daptomycin, E—erythromycin, FU—fusidic acid, CN—gentamicin, IMI—imipenem, LNZ—linezolid, MXF—moxifloxacin, MUP—mupirocin, F—nitrofuran, OX—oxacillin, P—penicillin, RD—rifampicin, TEC—teicoplanin, TE—tetracycline, SXT—trimethoprim */sulfamethoxazole, VA—vancomycin, LEV—levofloxacin, TGC—tigecycline, TOB—tobramycin.

As shown in Figure 2 and Table 2 (below), 89% and 79% of tested isolates were methicillin sensitive *S. aureus* isolates that were penicillin and ampicillin resistant, respectively. The high number of methicillin-sensitive isolates were actually penicillin-resistant phenotypes. Despite penicillin resistance, the high rates of antimicrobial susceptibility is a pattern of a resident lineages as shown below. The highly significant sensitivity pattern is indicated below for the rest of antimicrobials including full susceptibility to the following antibiotics: AUG (100%), DAP (100%), MUP (100%), IMI (100), TGC (100%), almost complete susceptibility to LNZ (99%), F (99%), CTX 95%, FOX 97.4%, OX (94.4%), TEC (99%), RD (97%), VA (99%), CN (97%), TE (93%), SXT (93%), and over 80% of isolates tested were susceptible to most of the drugs. Intermediate resistances were only seen in cases of antimicrobials LEV, Fu, and MFX. The rates of isolates intermediately susceptible for these drugs were 9%, 4%, and 1% only.

**Figure 3:**
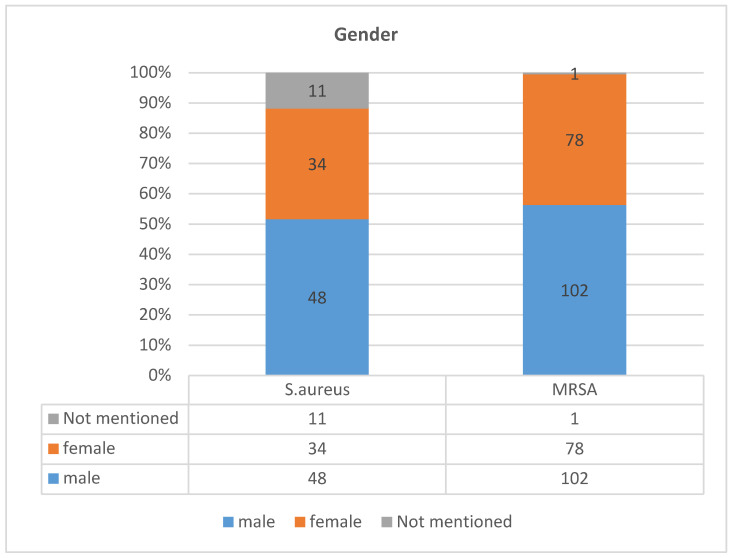
Gender differences in methicillin-resistant and susceptible *Staphylococcus aureus* distributions among men and women in the Ha’il region, Saudi Arabia.

**Figure 4:**
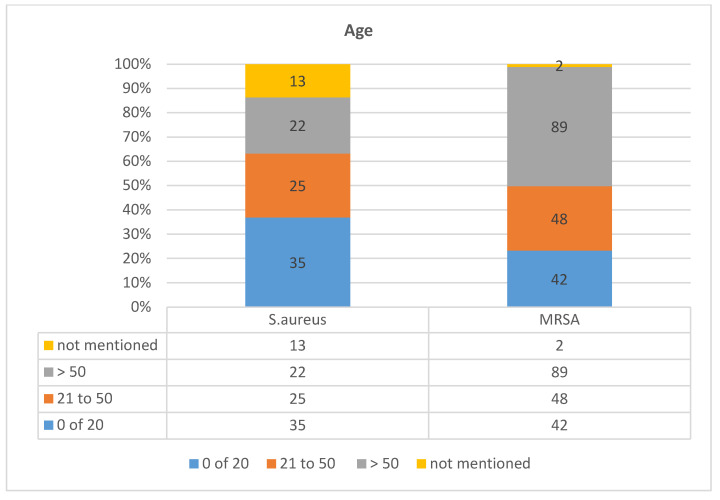
Age-specific distributions of methicillin-resistant and susceptible *Staphylococcus aureus* lineages in Ha’il region, Saudi Arabia.

Age- and gender-specific distribution profiles of *S. aureus* cutaneious infections showed consistent patterns, where MRSA dominated with an overall 66% (*n* = 181) of overall infections compared to 34% (*n* = 93) MSSA. In gender differences (Figure 3 and Table 3), MRSA infection in males was over 50% (37%, *n* = 102) compared to MSSA (17.5%, *n* = 48%). However, these were 12.4% (*n* = 34) and 28.4% (*n* = 78) for MSSA and MRSA infections in females, respectively. *Staphylococcus aureus* lineages MRSA and MSSA distributions were consistently increasing with increase in age for the former lineage and decreasing for the latter (Figure 4 and Table 4). The MRSA infections in age groups 0 to 20, 21 to 50, and over 50 years of age were 15% (*n* = 42), 17% (*n* = 48), and 32% (*n* = 89), respectively. However, the MSSA infections in the same age groups were 0 to 20 (13%, *n* = 35), 21 to 50 (9%, *n* = 25), and over 50 years of age (8%, *n* = 22), while 5% (*n* = 13) were missing gender information. This interesting pattern revealed an increasing MRSA infection with age with concomitant decreasing trend of MSSA infections across patients in the same age groups.

## 4. Discussion

The rapidly changing epidemiology of *S. aureus* leading to increased evolution of strains with enhanced virulence has been one the most significant public health and healthcare issues in this era. With increased human dynamics, new lineages constantly emerge crossing host barriers and making transmission dynamics between human—human, zoonotic and anthroponotic, and livestock transmissions such as poultry- and bovine mastitis-lineages. Consequently, wide-spread evolutionary changes are taking place in hospital strain profiles for the introduction and replacement by community-associated lineages. Middle Eastern countries, in particular Saudi Arabia, have been making significant developments as a global economic hub. This makes it imperative for understanding and monitoring local and global strain profiles associated with humans and livestock products.

In the Arabian Peninsula in general and Saudi Arabia specifically, MRSA became evident as a severe cause of serious health issues, with numerous studies reporting prevalence rates up to nearly 50% [49] and over [50]. In some instances, the prevalence and incidence rates are much higher often with serious consequences and poor patient outcomes. Despite enormous efforts in primary MRSA screening and containment protocols in place, surveillance reports on the profiles of these lineages in the Middle East are limited. In addition, under most common protocols and practices, the prevalence rate of MRSA shows high variation in regional distribution like in western (42%), central (32%), and eastern (27%) areas, respectively [51,52]. Thus, there is a serious paucity of high quality data on *S. aureus* across the region. In this study, of all 276 staphylococcal isolates, 183 (66%) were MRSA, which is quite high but falls well within prediction rates for the increasing frequencies since the previous report of 38% [53]. Usually, the rates are not constant, and they do differ with geographical distribution in different countries in the region. As the Kingdom of Saudi Arabia is a major economic country in the region and the world in addition to its holy Islamic sites annually visited by millions of people from all over the world, increasing rates of potential global lineages is well justified.

In the current study, the MRSA isolates showed an extreme resistance against imipenem (IMI), amoxicillin/clavulanic acid (AUG), cefotaxime (CTX), cefoxitin (FOX), and oxacillin (OX) at the rate of >82%. In addition, nearly all isolates were fully resistant to penicillin (P) at 99% and ampicillin (AMP) at 98%. This scenario is well known to be substantially associated with high morbidity and mortality due to severe hospital-associated methicillin-resistant *S. aureus* (HA-MRSA) in bloodstream and soft tissue infections. Mono-therapeutics with first-line antimicrobials as the glycopeptide vancomycin or the lipopeptide daptomycin are also associated with reduced susceptibilities and therapeutic failure, unless combined with first-line agents to improve β-lactam susceptibility in a seesaw effect [54]. Nevertheless, solid evidence exists against the use of beta-lactams either alone or in combination as they have been found to enhance MRSA virulence by the powerful universal gene-expression hub, the SarA gene family that code for proteins involved in quorum-sensing [55]. By virtue of being MRSA, the resistance reported here are not surprising as much as to the pattern of CA-MRSA resistance and the number of resistant isolates circulating.

We report on a high number of MRSA isolates susceptible to several non-beta-lactams often reaching to 100% susceptibility consistent with CA-MRSA pattern. These were tigecycline (TGC) 100%, linezolid (LNZ) 98.3% rifampicin (RD) 97.2%, teicoplanin (TEC) 97.2%, vancomycin (VA) 96%, levofloxacin (LE) 91%, tetracycline (TE) 89.3%, trimethoprim/sulfamethoxazole (SXT) 84.5%, and inducible macrolide (iMLS) 81%. This is in agreement with the previous studies that all MRSA isolates identified were susceptible to non-beta-lactams [56,57]. For this, vancomycin should be kept as the first choice for empiric treatment of MRSA with continued use of linezolid to be considered as the last resort. In line with previously conducted studies, the same trends have been reported by other authors [58,59]. However, in this study, high susceptibility was reported towards TGC, TE, SXT, and LE which is an improvement from previously reported resistances from the Middle East [60,61,62]. The changes reported in the spectrum of antimicrobial susceptibility of previously resistant patterns is potentially the outcome of stricter MRSA screening protocols in-pace and antimicrobial stewardship. This promising MRSA susceptibility indicated that rifampicin and sulfamethoxazole/trimethoprim could be a better empirical option in these regions. As this study was from Ha’il hospitals in the remote northern regions of Saudi Arabia, the risk of resistance transfer among Gram-positives is low and there is no report yet on the two globally emerging rifampicin resistant S. epidermidis lineages found in 24 countries that concomitantly reduce susceptibility to vancomycin and teicoplanin [63]. Thus, the full resistance to beta-lactam and susceptibility to non-beta-lactam antibiotics reported here is the typical pattern of CA-MRSA lineages [32]. This is further supported by the high number of these isolates from young, otherwise healthy patients, which is one property of this lineage. This makes it imperative for future large-scale surveillance of all outpatients and inpatients in a downstream molecular surveillance to identify sequence-type, clonal complex, pvl gene, and resistance cassette types [64]. Moreover, our isolates have shown more than 99% susceptibility towards linezolid (LNZ), nitrofuran (F), cefotaxime (CTX), cefoxitin (FOX), oxacillin (OX), teicoplanin (TEC), rifampicin (RD), vancomycin (VA), gentamicin (CN), tetracycline (TE), and sulfamethoxazole/trimethoprim (SXT).

About 98.9% of methicillin-susceptible *Staphylococcus aureus* in this study were penicillin-resistant. However, they were 100% susceptible in vitro to the potentiated amoxicillin/clavulanic acid (AUG) and other non-beta-lactams such as daptomycin (DAP), mupirocin (MUP), imipenem (IMI), tigecycline (TGC). The increased use of clavulanate potentiated amoxicillin may have enriched penicillin resistance in methicillin-susceptible phenotypes in the present study. This observation has been previously reported in increased childhood nasal colonization of penicillinase producing methicillin-susceptible *S. aureus* [65]. Similarly, an epidemic outbreak of Panton–Valentine leukocidin (PVL) positive methicillin-susceptible *S. aureus* (MSSA) was reported in a maternity hospital that was initiated by postpartum mastitis and neonatal skin infections [66]. Due to biased sequencing of MRSA lineages, MSSA strains frequently receive less attention albeit they are associated with serious infections in humans. Genome sequencing of a highly virulent yet pan-susceptible MSSA isolate from a fatal case of sepsis and bacteraemia in a dengue patient revealed a novel combined genotype (t091/ST2990). A β-lactamase plasmid, staphylococcal enterotoxin, and enterotoxin-like genes were identified in addition to phylogenetic evidence of common ancestry with the European MRSA clone [67]. In the current study, higher susceptibility was seen for MSSA than MRSA to both beta-lactam and non-beta lactam antibiotics indicating the usefulness of continued surveillance in identifying susceptibility profiles. A 15-year retrospective surveillance at two tertiary care institutions in Boston, MA with 31,753 adult inpatients revealed *S. aureus* infection declined from 2000 to 2014 by 4.2%, due to an annual decline in MRSA of 10.9%. Consequently, penicillin-susceptible *S. aureus* (PSSA) increased by 6.1% annually, while the rates of methicillin-susceptible penicillin-resistant *S. aureus* (MSSA) did not change (10% to 11%; *p* value 0.43). Furthermore, 3/14 MSSA and 2/21 PSSA isolates arose from the loss of resistance-conferring genes. The decline in *S. aureus* infections has been accompanied by a shift toward increased antibiotic susceptibility [68]. Thus, constant surveillance and resistance programs are critical for the evolution of susceptible strains, empiric therapy, and combating invasive *S. aureus*.

Our result depicts high prevalence of MRSA among elderly patients with underlying risks aged >50 years (32%). Past studies presented considerably variable data regarding the distribution of MRSA in varied age groups. There are ample studies from Saudi Arabia and other Middle Eastern countries that showed MRSA common occurrence in elderly patients [56,69], and this is attributed to the common risk factors including age, co-morbidities, and long hospital stays. However, the frequency of infection reported in this study among otherwise healthy and mostly young patient groups, 0–20 years (15%) and 21–48 years (17%) with no underlying risk of comorbidity or hospitalization, is consistent with established host properties of CA-MRSA [70]. These were mostly male patients harboring 37% (*n* = 102 of 276) of total isolates followed by female patients at 28.4% (*n* = 78 of 276) with the ratio of 1.3:1. This may be attributed to the local differences in more male outdoor socializations than females in addition to the lack of hygiene practice [69,71,72,73].

## 5. Conclusions

Despite enormous efforts and strict MRSA screening in pre-hospital admissions, the rates of the *S. aureus* lineages, particularly in men and senior patients with underlying risk, are still the highest. This is potentially due to increased use of beta-lactams known to enhance *S. aureus* virulence. Intriguingly, most of the isolates had CA-MRSA patterns with high susceptibility to non-beta-lactams and increased prevalence in young and otherwise healthy individuals. However, almost all MSSA phenotypes identified in this study were only penicillin-resistant. Taken together, we report on three *S. aureus* lineages, each with unique evolutionary dynamics and host-specificity, i.e., the decreasing trend of MSSA by age with the concomitant increase and sub-clonal differentiation into HA-MRSA in seniors and CA-MRSA in young and otherwise healthy patients. These profiles strongly imply age-specific evolutionary selection of these strains from a resident MSSA ancestor. Future vertical studies should focus on the surveillance of invasive CA-MRSA rates and phenotypes.

## Figures and Tables

**Table 1:** Percentage sensitive, resistant, and intermediate methicillin-resistant *Staphylococcus aureus* isolates across 26 different antimicrobials in Ha’il, Saudi Arabia.

	AUG	AMP	CTX	FOX	CIP	CD	DAP	E	FU	CN	IMI	LNZ	MXF	MUP	F	OX	P	RD	TEC	TE	SXT	VA	iMLS	LEV	TGC	TOB
Sensitive	16.9	1.4	14.4	13.3	81.1	82.1	97.9	64.6	51.7	65.7	18.2	98.3	87.0	96.6	96.7	12.8	1.1	97.2	97.2	89.3	84.5	96.1	81.8	90.9	100.0	54.5
Intermediate								0.6	6.7	1.1		0.6	2.8							1.1		0.6				
Resistant	83.1	98.6	85.6	86.7	18.9	17.9	2.1	34.8	41.6	33.1	81.8	1.1	10.2	3.4	3.3	87.2	98.9	2.8	2.8	9.6	15.5	3.4	18.2	9.1		45.5
*n*	148	142	146	180	148	179	146	178	178	181	148	179	177	145	180	179	179	178	179	178	181	179	33	33	33	33

**Table 2:** Percentage susceptible, resistant, and intermediate methicillin-susceptible *Staphylococcus aureus* isolates across 26 different antimicrobials in Ha’il, Saudi Arabia.

	AUG	AMP	CTX	FOX	CIP	CD	DAP	E	FU	CN	IMI	LNZ	MFX	MUP	F	OX	P	RD	TEC	TE	SXT	VA	LEV	TGC	TOB
Sensitive	100.0	21.1	94.7	97.4	87.2	82.2	100.0	60.8	62.3	97.1	100.0	98.7	89.2	100.0	98.7	94.4	11.1	97.1	98.7	93.3	93.2	98.7	83.9	100.0	67.9
Intermediate									3.8				1.4										9.7		
Resistant		78.9	5.3	2.6	12.8	17.8		39.2	34.0	2.9		1.3	9.5		1.3	5.6	88.9	2.9	1.3	6.7	6.8	1.3	6.5		32.1
*n*	39	38	38	39	39	73	39	74	53	69	39	75	74	39	75	54	54	68	75	75	74	75	31	36	28

**Table 3:** Gender-specific distributions of *Staphylococcus aureus* lineage infections in Ha’il region, Saudi Arabia.

Gender	MSSA	MRSA
male	48 (17.5)	102 (37)
female	34 (12.4)	78 (28.4)
Not mentioned	11	1
total	93 (34%)	181 (66%)

**Table 4:** Patterns of *Staphylococcus aureus* lineage infections across different age-groups in Ha’il region, Saudi Arabia.

Age	0 of 20	21 to 50	>50	Not Mentioned	Total
MSSA	35 (13%)	25 (9%)	22 (8%)	13 (5%)	95 (34.4)
MRSA	42 (15%)	48 (17%)	89 (32%)	2	181 (65.5%)

## Data Availability

Data are contained within the article.
